# Self-control is associated with health-relevant disparities in buccal DNA-methylation measures of biological aging in older adults

**DOI:** 10.1186/s13148-024-01637-7

**Published:** 2024-02-08

**Authors:** Y. E. Willems, A. deSteiguer, P. T. Tanksley, L. Vinnik, D. Fraemke, A. Okbay, D. Richter, G. G. Wagner, R. Hertwig, P. Koellinger, E. M. Tucker-Drob, K. P. Harden, Laurel Raffington

**Affiliations:** 1 Max Planck Research Group Biosocial – Biology, Social Disparities, and Development, Max Planck Institute for Human Development, Lentzeallee 94, 14195 Berlin, Germany; 2 Population Research Center, The University of Texas, Austin, USA; 3 School of Business and Economics, Economics Fellow, Tinbergen Institute, Amsterdam, The Netherlands; 4 Amsterdam Neuroscience, Complex Trait Genetics, Vrije Universiteit Amsterdam, Amsterdam, The Netherlands; 5 Department of Economics, School of Business and Economics, Vrije Universiteit Amsterdam, Amsterdam, The Netherlands; 6 Max Planck Institute for Human Development, Berlin, Germany; 7 SHARE Berlin Institute GmbH, Berlin, Germany; 8 Department of Education and Psychology, Freie Universität Berlin, Berlin, Germany; 9 German Socio Economic Panel Study (SOEP), Berlin, Germany

**Keywords:** Self-control, DNA-methylation, Pace of aging, Biological aging, Health, Life span

## Abstract

Self-control is a personality dimension that is associated with better physical health and a longer lifespan. Here, we examined (1) whether self-control is associated with buccal and saliva DNA-methylation (DNAm) measures of biological aging quantified in children, adolescents, and adults, and (2) whether biological aging measured in buccal DNAm is associated with self-reported health. Following preregistered analyses, we computed two DNAm measures of advanced biological age (principal-component PhenoAge and GrimAge Acceleration) and a DNAm measure of pace of aging (DunedinPACE) in buccal samples from the German Socioeconomic Panel Study (SOEP-G[ene], *n* = 1058, age range 0–72, *M*_age_ = 42.65) and saliva samples from the Texas Twin Project (TTP, *n* = 1327, age range 8–20, *M*_age_ = 13.50). We found that lower self-control was associated with advanced biological age in older adults (PhenoAge Acceleration *β* = − .34, [− .51, − .17], *p* < .001; GrimAge Acceleration *β* = − .34, [− .49, − .19], *p* < .001), but not young adults, adolescents or children. These associations remained statistically robust even after correcting for possible confounders such as socioeconomic contexts, BMI, or genetic correlates of low self-control. Moreover, a faster pace of aging and advanced biological age measured in buccal DNAm were associated with self-reported disease (PhenoAge Acceleration: *β* = .13 [.06, .19], *p* < .001; GrimAge Acceleration: *β* = .19 [.12, .26], *p* < .001; DunedinPACE: *β* = .09 [.02, .17], *p* = .01). However, effect sizes were weaker than observations in blood, suggesting that customization of DNAm aging measures to buccal and saliva tissues may be necessary. Our findings are consistent with the hypothesis that self-control is associated with health via pathways that accelerate biological aging in older adults.

**Supplementary Information:**

The online version contains supplementary material available at 10.1186/s13148-024-01637-7.

## Introduction

Self-control is a dimension of personality that encompasses the ability to delay gratification, inhibit behavioral impulses, and regulate the expression of emotions. Self-control has been proposed to be a key behavioral mediator of both environmental and genetic risk factors for aging-related morbidity and mortality [10, 11, 17, 39, 40, 54]. Individual differences in self-control arise early in the life course and are associated with myriad health-relevant behaviors and exposures, including sleep, substance use, nutrition, exercise, and socioeconomic attainments [8, 24, 37, 60]. These behaviors and exposures have, in turn, been associated with a faster pace of biological aging across multiple physiological systems [42, 43, 63]. Little work, however, has directly investigated whether self-control is related to biological aging, which describes the gradual decline in system integrity across tissues and organs that occurs with advancing chronological age [27, 32].

Recently, DNA-methylation (DNAm) measures have been developed to quantify processes of biological aging. DNAm is a stable epigenetic marker that underpins the lifelong maintenance of cellular identity and a dynamic developmental process that changes with age and environmental inputs [33]. Specifically, DNAm measures have been developed to quantify *accelerated biological age* and mortality risk (e.g., GrimAge and PhenoAge Acceleration [30, 34]; as well as the *pace of aging* across 18 physiological systems measured repeatedly in the same people (i.e., DunedinPACE, [5].

Recent research based on blood samples suggests that lower self-control is associated with accelerated biological age and earlier mortality as indicated by GrimAge Acceleration in 17–50 years old adults [21, 29]. Moreover, in a five decade prospective study, children with lower self-control later experienced a faster pace of aging in midlife as indicated by analyses of physiological biomarkers [53]. As adults, they were also less attentive to practical health information, less consistent in implementing positive health behaviors, and exhibited less positive expectancies about aging. Additionally, those individuals’ self-control in midlife was associated with their pace of aging even after accounting for their self-control in childhood. This suggests that self-control may exert differential influences on aging processes at different points in the life span. It remains unexplored when in the life course associations of self-control with biological aging may become visible; it could take decades until the aging consequences of low self-control arise. DNAm quantifications of biological aging in cohorts of varying ages can help address this question.

While DNAm measures of biological aging are typically developed using blood DNA, buccal and saliva DNA are also commonly collected, particularly in younger cohorts. Buccal and saliva can be sampled via postal kits and this procedure has substantially higher participation rates than blood sampling (e.g., saliva 72% vs. blood 31%, [19]. Previous findings provide evidence for good saliva-blood cross-tissue correspondence. Blood, saliva and buccal are partially composed of the same cell types: Blood samples consist of 100% immune cells, saliva in children consist of approximately ~ 35% epithelial cells and ~ 65% immune cells [38], and buccal cells in adults consist of ~ 80% epithelial cells and ~ 20% immune cells [59, 65]. While statistical corrections for people’s cell composition are common, immune cell DNAm may be particularly sensitive to early life exposures and aging-related inflammatory processes that can affect multiple tissues, including neurons [7]. Additionally, DNAm measures computed in both blood and saliva tissues from the same persons show high cross-tissue rank-order stability [47, 48]. More research is needed to assess the applicability of blood-based DNAm measures particularly to buccal tissue, for which cross-tissue rank-order stability appears to be lower than saliva [50].

Here, we examined (1) whether self-control is associated with buccal and saliva DNAm measures of biological aging (DunedinPACE, GrimAge Acceleration, and PhenoAge Acceleration) quantified in children, adolescents, and adults, and (2) whether biological aging measured in buccal DNAm is associated with self-reported health. Buccal DNA was collected from participants in the German Socioeconomic Panel Study (SOEP-G[ene], *n* = 1058, age range 0–72, *M*_age_ = 42.65) and saliva DNA from participants in the Texas Twins Project (TTP, *n* = 1327, 8–20, *M*_age_ = 13.50). We further tested whether associations differed by chronological age and remained after statistical correction for socioeconomic contexts, body mass index, and smoking, which are commonly associated with DNAm measures of biological aging [46–48], as well as a genetic correlate of low self-control (i.e., a polygenic score of externalizing problems, [26]. We employed principal-component-based versions of PhenoAge and GrimAge Acceleration to increase reliability [23]. We preregistered our study and highlight where our measures or analyses deviated from our plan (https://osf.io/5sejf, Additional file 1: Table S1). We report standardized beta parameters with 95% confidence intervals. We report nominal *p* values taking *p* < .05 as a threshold, and note if results remain significant after Benjamini–Hochberg False-Discovery-Rate method correction (FDR, [6]).

## Results

1. Lower self-control is associated with accelerated biological age in buccal tissue from older participants, but not younger adults, adolescents, or children.

**Fig. 1:**
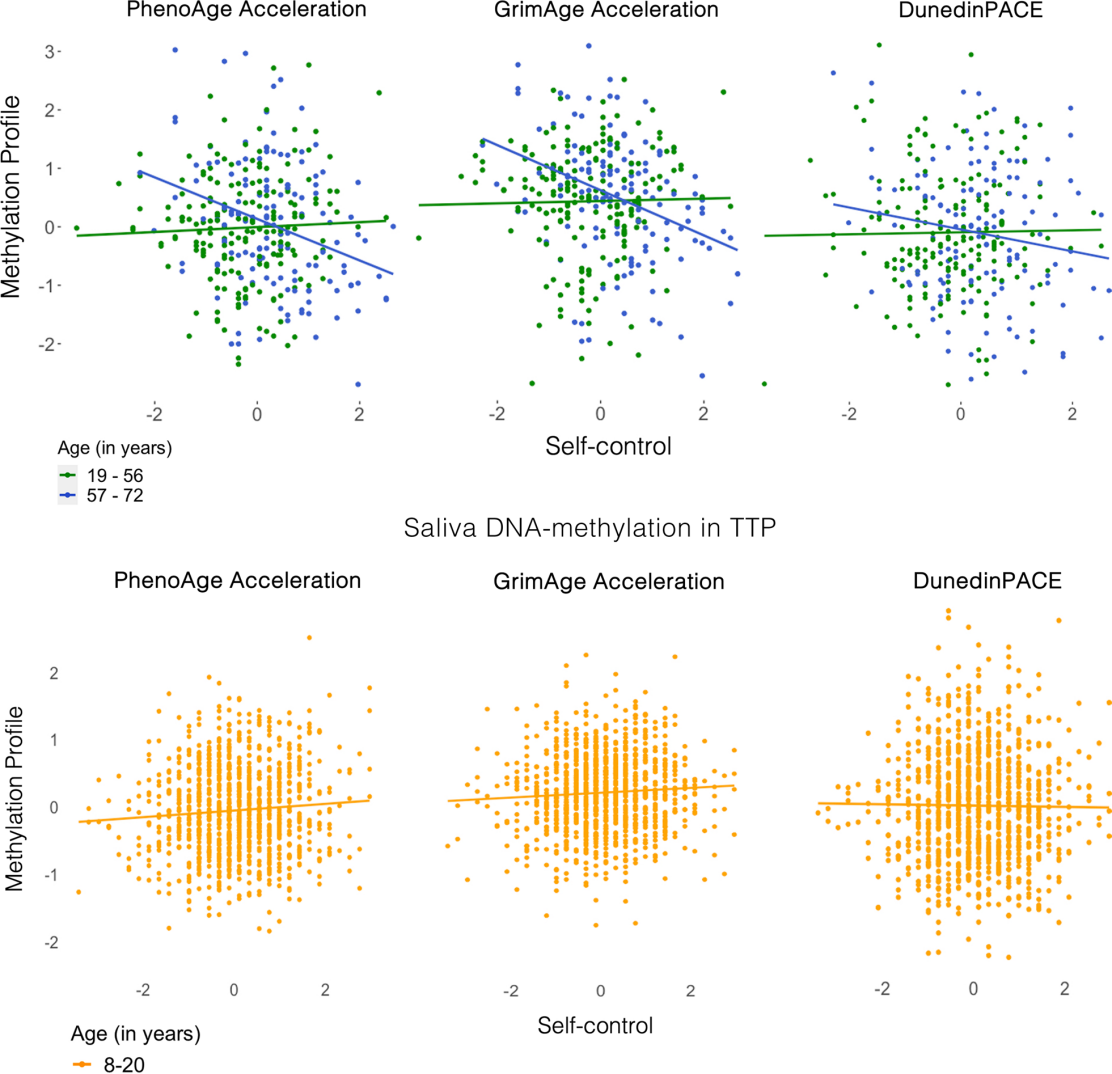
Associations between self-control and DNA-methylation measures of biological aging. *Note* The age group split presented in our findings serve to illustrate the significant interactions, as the regression analyses employ age as a continuous variable. DNAm-aging measures and self-control are scaled, and principal-component-based versions of PhenoAge and GrimAge Acceleration were used. Self-control was measured with the BTS in SOEP-G and with the grit scale in TTP. See Additional file 1: Fig. S1 for associations of DNAm with attention problems and impulsivity measures in TTP

First, we examined whether self-control was associated with DNAm measures of biological aging. In SOEP-G, we found that lower self-control (as measured by the Brief Tangney Self-control Scale [56], *n* = 333) was associated with more advanced PhenoAge and GrimAge Acceleration but not with a faster DunedinPACE (PhenoAge *β* =  − .13 [− .25, − .01], *p* = .03; GrimAge *β* =  − .15 [− .26, − .04], *p* = .01; DunedinPACE *β* =  − .06 [− .17, .04], *p* = .25). These associations did not survive FDR correction for multiple comparisons. In TTP, children and adolescents’ self-control was not significantly associated with saliva DNAm measures of biological aging (see Fig. 1, Additional file 1: Tables S2 and S3).

Next, according to our pre-registered analysis plan, we examined whether the association between self-control and DNAm measures of biological aging differed by chronological age in SOEP-G. We regressed measures of biological aging on self-control, chronological age, and the interaction between self-control and age. We found that the association between self-control with PhenoAge and GrimAge Acceleration, but not DunedinPACE, was significantly moderated by chronological age (PhenoAge *β* =  − .20 [− .34, − .05], *p* < .01; GrimAge *β* =  − .17 [− .28, − .06], *p* < .01; DunedinPace *β* =  − .10 [− .24, .03], *p* = .14). These interaction terms remained significant after FDR correction. Accordingly, lower self-control was associated with accelerated biological age in older participants.

To further characterize this age interaction, we stratified participants into older and younger participants by mean split (*M*_age_ = 57.02). Among older participants (aged 57–72 years, *n* = 140), lower self-control was associated with more advanced PhenoAge and GrimAge Acceleration (PhenoAge *β* =  − .34, [− .51, − .17], *p* < .001; GrimAge *β* =  − .34, [− .49, − .19], *p* < .001; see Fig. 1). In contrast, among younger participants (aged 19–56, *n* = 193), self-control was not associated with PhenoAge or GrimAge Acceleration (PhenoAge *β* = .06, [− .09, .21], *p* = .45; GrimAge *β* = .03, [− .19, .12], *p* = .66). The association between self-control and DunedinPACE was not statistically significant in younger or older participants (younger *β* = .02 [− .14, .17], *p* = .84; older *β* =  − .17, [− .35, .00], *p* = .06; see Fig. 1).

We have previously found that socioeconomic disadvantage is associated with accelerated buccal PhenoAge and GrimAge and a faster DunedinPACE in SOEP-G [50] as well as a faster saliva DunedinPACE, but not accelerated PhenoAge or GrimAge, in a subsample of TTP children [47, 48]. Therefore, we tested whether associations of self-control and DNAm measures of biological aging were accounted for by socioeconomic contexts.

We found that the association of self-control with PhenoAge and GrimAge Acceleration remained statistically significant after controlling for socioeconomic contexts in SOEP-G (see Additional file 1: Table S4). In contrast to a previous analysis of *n* = 600 TTP children, which found an association only with DunedinPACE, socioeconomic disadvantage was also associated with accelerated GrimAge in the current sample of *n* = 1327 TTP children, even after statistical correction for smoking, BMI, and pubertal timing (*β* =  − .13 [− .19, − .07], *p* < .001, Additional file 1: Table S5).

Additionally, associations of self-control with PhenoAge and GrimAge Acceleration in SOEP-G remained statistically significant after controlling for BMI, and genetic correlates of low self-control (see Additional file 1: Tables S6 and S7). (There were no self-reported smokers in the subsample that had data available on both self-control and DNAm measures.) Risk preference, which consisted of just one response item and was weakly correlated with the Brief-Tangney Self-control scale (*r* = .07, *p* < .05), was not associated with DNAm biological aging measures (see Additional file 1: Table S8).

In sum, lower self-control was associated with accelerated biological age in older participants, but not younger adults, adolescents, or children.

(2) A faster pace of aging and accelerated biological age measured in buccal DNAm are associated with worse self-reported health.

**Fig. 2:**
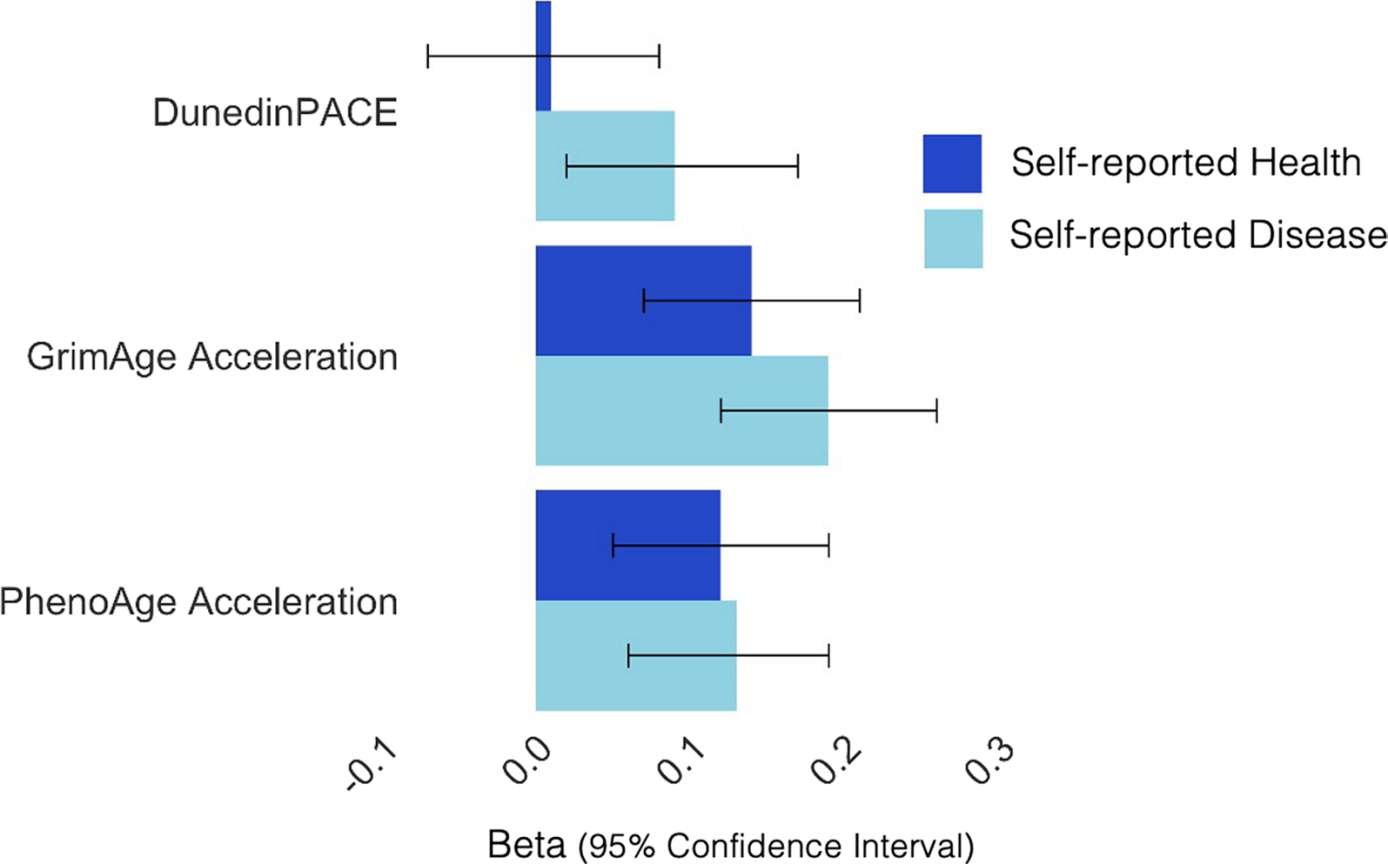
Standardized associations between buccal DNAm measures of biological aging and health in SOEP-G (principal-component-based versions of PhenoAge and GrimAge Acceleration were used). Higher levels of self-reported disease indicate worse health. For illustration purposes, self-reported health was reverse coded such that higher levels also reflect worse health

Next, non-preregistered analyses evaluated whether buccal DNAm measures of biological aging were associated with self-reported disease and self-reported health in SOEP-G (*n* = 797). These analyses focused on SOEP-G as the TTP consists of children and adolescents that are generally in good health. The moderate-to-strong correlation coefficient (*r* =  − .64, 95% CI =  − .67 to − .61, *p* < .001) between self-reported disease and self-reported general health indicates that both measures are tapping into a common domain (i.e., health), but nevertheless capture unique components of health and well-being. While self-reported disease assesses people’s current state of physical disease (higher scores indicate higher disease burden), self-reported health assesses whether people can live their life without any limitations due to physical and/or mental health problems (higher scores indicate better health; scale is reverse coded in Fig. 2).

We found that accelerated biological age and faster pace of aging were significantly associated with more self-reported disease (PhenoAge Acceleration: *β* = .13 [.06, .19], *p* < .001; GrimAge Acceleration: *β* = .19 [.12, .26], *p* < .001; DunedinPACE: *β* = .09 [.02, .17], *p* = .01). Accelerated biological age, but not pace of aging, was also associated with worse health, as indicated by self-reported general physical and mental health (See Fig. 2; PhenoAge Acceleration: *β* =  − .12 [− .19, − .05], *p* < .001; GrimAge Acceleration: *β* =  − .14 [− .21, − .07], *p* < .001; DunedinPACE: *β* =  − .00 [− .08, .07], *p* = .967). These results remained significant after FDR correction. There were no significant interaction effects with age (see Additional file 1: Table S9).

Next, we tested whether associations of buccal DNAm measures of biological aging with health were statistically accounted for by socioeconomic contexts, BMI, and smoking. We found that the association between DunedinPACE and self-reported disease severity was accounted for by BMI and socioeconomic contexts (see Additional file 1: Table S10 and S11). Associations between PhenoAge and GrimAge Acceleration with self-reported disease severity and health remained statistically significant after accounting for BMI, smoking and socioeconomic contexts (see Additional file 1: Table S10 and S11).

Finally, we examined whether buccal DNAm measures of biological aging statistically accounted for associations of self-control with health (*n* = 333). GrimAge Acceleration statistically accounted for 9% of the associations between self-control and self-reported disease severity and health, respectively, in the total sample (indirect effect *β* =  − .02, [− .04, − .00], *p* = .03, see Table 1). We repeated these analyses for older participants only, for whom self-control was associated with PhenoAge and GrimAge Acceleration (see above). Among older participants, GrimAge Acceleration statistically accounted for 26% of the association between self-control and self-reported disease severity (indirect effect *β* =  − .07, [− .14, − .01], *p* = .03, see Additional file 1: Table S12). These indirect pathways were significant at the nominal *p* value, but not survive FDR correction. Importantly, these mediation analyses are based on cross-sectional data and thus do not allow for causal inference.

**Table 1:** Indirect path estimates of DNA-methylation measures of biological aging statistically accounting for associations of self-control with health

	Accelerated biological age						Pace of aging		
	PhenoAge acceleration			GrimAge acceleration			DunedinPACE		
	*B*	95% CI	*p*	*B*	95% CI	*p*	*B*	95% CI	*p*
*Self-control → disease severity*									
Total effect	− **.22**	**[**− **.28,** − **.16]**	** < .001**	− **.22**	**[**− **.28,** − **.16]**	** < .001**	− **.22**	**[**− **.28,** − **.16]**	** < .001**
Direct effect	− **.21**	**[**− **.27,** − **.15]**	** < .001**	− **.20**	**[**− **.27,** − **.14]**	** < .001**	− **.22**	**[**− **.28,** − **.15]**	** < .001**
Indirect effects	− .01	[− .03, .02]	.09	− **.02**	**[**− **.04,** − **.00]**	**.03**	− .01	[− .02, 01]	.47
*Self-control → health*									
Total effect	**.22**	**[.15, .29]**	** < .001**	**.22**	**[.15, .29]**	** < .001**	**.22**	**[.15, .29]**	** < .001**
Direct effect	**.21**	**[.14, .28]**	** < .001**	**.21**	**[.14, .27]**	** < .001**	**.22**	**[.15, .29]**	** < .001**
Indirect effects	.01	[− .00, .03]	.10	**.02**	**[.00, .03]**	**.04**	− .00	[− .01, .01]	.85

Bold estimates significant at the *p* <.05 level

## Discussion

We examined (1) whether self-control is associated with buccal and saliva DNAm measures of biological aging quantified in children, adolescents, and adults, and (2) whether biological aging measured in buccal DNAm is associated with self-reported health. First, we found that lower self-control was associated with more advanced biological aging in older participants (57–72 years), but not young adults, adolescents or children. The association between self-control with PhenoAge and GrimAge Acceleration in older participants remained statistically significant after controlling for socioeconomic contexts, BMI, smoking, and genetic correlates of self-control. Second, our results indicated that both advanced biological age and a faster pace of aging measured in buccal DNAm were associated with more self-reported disease. While the association between DunedinPACE and self-reported disease severity was accounted for by BMI, smoking and socioeconomic contexts, PhenoAge and GrimAge Acceleration were related to self-reported disease after accounting for BMI, smoking and socioeconomic status. PhenoAge and GrimAge Acceleration were also related to self-reported health, over and above covariate control. Our finding that DunedinPACE is only related to our disease measure but not our health measure might indicate it is more sensitive to measures of physical than mental health.

Thus, despite low-to-moderate cross-tissue correspondence across blood and buccal measures (PhenoAge Accel. *r* = .25, GrimAge Accel. *r* = .48, DunedinPACE. *r* = .31; [50], buccal DNAm measures of biological aging appear to capture aging processes relevant to disease and health. But, effect sizes were weaker than observations in blood (GrimAge and health in buccal *β* = .10–.20 versus blood *β* = .10–.50, [15, 16, 25, 31, 36]. Thus, customization of DNAm aging measures to buccal tissues may be necessary to maximize their utility.

Collectively, our findings are consistent with the hypothesis that self-control is associated with health via pathways that accelerate biological aging in midlife and older age. Among older SOEP-G participants, buccal GrimAge Acceleration statistically accounted for 26% of the association between self-control and self-reported disease severity and health. Among younger SOEP-G and Texas Twin participants, self-control was not associated with biological aging. The effects of self-control-related behaviors on biological aging are likely to accumulate over time, thus, the aging consequences of low self-control may not be visible in the first few decades of life, when people are generally healthy. Moreover, findings from a prospective birth cohort study suggest that self-control in childhood compared to self-control in midlife shows lower rank order stability and may exert independent influences on later life aging [53].

We acknowledge limitations. First, our study is based on cross-sectional data and can therefore not make inferences about the direction of the effects between self-control, biological aging, and health. We cannot disentangle whether differences in self-control cause accelerated aging and worse health or, in reverse, worse health causes lower self-control and advanced biological aging. Similarly, age differences in associations between self-control and biological aging could arise from developmental differences or cohort effects related to generational differences (e.g., environmental toxicants, social structures). Second, our findings are likely to be somewhat tissue specific. It is possible, for example, that self-control is associated with the pace of aging in younger samples when DNAm is quantified in blood rather than saliva. In order to take full advantage of buccal and saliva DNA samples, DNAm algorithms developed in these tissues may be needed. Third, our measures of self-control were limited and differed between the two cohorts. Future research measuring self-control across informants, ages, and situations is important to tap into the broader range of real-world capacities that comprise this umbrella construct.

In conclusion, we find that self-control is associated with buccal DNA-methylation measures of biological aging in midlife and older adulthood in a health-relevant manner. If the cross-sectional findings reported here are found to be causal, then interventions that are successful in increasing self-control might extend the health span [18]. Alternatively, people’s proximate environments can be manipulated to put less demand on individual self-control behaviors [52].

## Methods

### Participants

#### SOEP-G

The Socioeconomic Panel (SOEP) is an ongoing population-based, multi-generational survey study. Parts of the SOEP are the “SOEP core” and the “SOEP-Innovation Sample (SOEP-IS), which are two independent random samples of German Households. The SOEP core consists of a broad set of standard survey questions on socioeconomic and sociodemographic background, SOEP-IS supplements this by incorporating data gathered through special questions and experimental modules. In total, SOEP-IS includes 6,576 participants, who were invited to participate in buccal DNA genotyping as part of the “gene subsample” (SOEP-G; [28]). In total, there are polygenic indices available for *n* = 2,063 adults (*M*_age_ = 56.13, SD_age_ = 18.72, 54% female), with 98% of participants showing high genetic similarity to European reference groups (see [28]).

Based on the availability of funds, residual frozen DNA samples of *n* = 1128 of the SOEP-G sample were selected for DNA-methylation analyses. The inclusion criteria were as following: (1) samples from children and adolescents with residual DNA samples holding at least 50 ng of DNA, (2) adults with extending age distribution past 18 years, that had at least 250 ng of DNA left, had a call rate of at least 0.975, and did not have participating children in the dataset to maximize number of households, and (3) match between genetic sex and self-reported sex (see [49] for more details). This resulted in the availability of DNA-methylation data for *n* = 1058 participants (*M*_age_ = 42.42, SD_age_ = 21.17, 58% female), for whom polygenic scores are also available (see above).

#### TTP

The Texas Twin Project (TTP) is an population-representative longitudinal study investigating children and adolescents in the greater metropolitan areas of Austin, Texas [20]. It has polygenic and DNAm data available for *n* = 1327 children and adolescents (*M*_age_ = 13.50, SD_age_ = 3.10, 48% females, 34.6% monozygotic twins, 58.9% dizygotic twins). Participants self-identified as White (59.5%), Hispanic/Latinx-only (10.7%), Black/African-American (10.4%), Asian (8.5%), and Hispanic/Latinx-White (7.8%).

## Measures

Measures are described in Table 2 and include description of the deviation from our preregistration if applicable. Descriptives are presented in Table 3.

**Table 2:** Description of measures

	Measures	
	SOEP-G *n* = 1058 Age range 0–72 *M*_age_ = 42.65 DNAm in Buccal Germany	TTP *n* = 1327 Age range 8–20 *M*_age_ = 13.50 DNAm in Saliva United States
(1) Self-control	*The Brief Tangney Self-Control Scale:* Consists of 13 self-report items on a 5-point Likert scale [56]. Example questions are: “I am good at resisting temptation” and “I have a hard time breaking bad habits.” A mean score was created based on the 13 items, with a higher overall mean score indicating higher self-control	*The Impulsivity and Sensations Seeking Scale:* We assessed impulsivity and sensation seeking with the Zuckerman–Kuhlman–Aluja Personality Questionnaire (ZKA-PW, [67]. This self-reported scale consists of 8 items measuring impulsivity and 11 items measuring sensation seeking, including items such as “I’m an impulsive person” and “I usually think about what I am going to do before doing it.” We created a mean score, with higher scores reflecting more impulsivity and sensation seeking
	*Risk preference:* We assessed risk preference or aversiveness with one item where participants are asked to rate themselves on a 11-point scale on the following question: “In general, are you someone who is willing to take risks or do you try to avoid risks?”. We recoded the scale such that higher scores reflect more risk aversiveness [2]	*The Attention Problems scale:* We used the 11 items of the attention problems scale of the Child Behavior Checklist [1]. Children filled in questions such as “I fail to finish things that I start,” “I can’t sit still,” on a 3-point scale. A sumscore was created, with higher scores reflecting more attention problems We preregistered to use the ASEBA Self-Control Scale, but the items required for this scale were not available. We therefore used the ASEBA-Attention problem scale, which overlaps in 4 items with the ASEBA-Self-Control Scale [64]
	Our preregistration included the Impulsivity and Patience scale (IPS, [62], but the Cronbach alpha of this scale was not sufficient (*Cronbach α* = .39) unlike the Brief Tangney Self-Control scale (*Cronbach α* = . 76). Thus, the Impulsivity and Patience scale was dropped from analyses	*Grit:* We used the Short Grit Scale (SGS) which is a self-report scale assessing diligence and grit with an 8-item questionnaire developed by Duckworth & Quinn [12]. It includes self-reported items on a 5-point scale with questions such as “new ideas and projects sometimes distract me from previous ones” and “setbacks don’t discourage me.” We created an overall sum score, with higher scores indicating more grit
(2) DNAm measures of aging-related health^a,b^	*DunedinPACE* was developed in the Dunedin Study birth cohort and is based on analyses of within-person change in 18 physiological markers measured repeatedly at age 26, 32, 38 and 45. It is an extension of the DunedinPoAm pace of aging which was based on a 12-year period, while DunedinPace is based on 20 years of follow-up [5, 13]. Briefly, DunedinPACE was developed using a subset of EPIC array probes that were also included on Illumina’s earlier 450 k array, showing to have higher test–retest reliability [55]. Subsequently, elastic-net regression analyses was applied to fit Pace of Aging to DNAm data to blood samples collected when participants were 45 years, resulting in a 173-CpG algorithm. DunedinPACE was calculated based on the algorithm published by Belsky et al. [5]	
	*GrimAge* is a DNAm measure developed on a set of physiological indicators using machine learning analyses and DNAm algorithms to predict morbidity and mortality. GrimAge signifies the age in years at which average mortality risk in the Framingham Heart Study Offspring cohort matches actually predicted mortality risk. We used DNAm principal components when computing GrimAge to increase reliability [23], and created GrimAge Acceleration by residualizing GrimAge for chronological age. We preregistered to use GrimAge version 2, but the code to calculate this score is not yet publicly available	
	*PhenoAge* is modeled based on physiological markers and chronological age and subsequently applied to a new sample modeled from DNA methylation to derive a final DNA methylation clock [30]. It represents the age in years at which average mortality risk in NHANES III matches the mortality risk predicted by the PhenoAge algorithm. We used DNAm principal components when computing PhenoAge to increase reliability [23], and created PhenoAge Acceleration by residualizing PhenoAge for chronological age	
(3) Socioeconomic contexts	*Family-level socioeconomic contexts* were indexed by an average *z*-score including household income (equivalent net income) from different resources such as employment, child support, unemployment benefits, and pensions corrected for the number of people living in the household) and educational attainment (the highest degree obtained by any individual in the household in number of educational years + additional occupational training years) corrected for the number of people living in the household	*Family-level socioeconomic contexts* In line with earlier studies using the TTP data [14], we computed a socioeconomic composite as the average of standardized parent educational attainment and standardized household income Initially, we preregistered a broad socioeconomic disadvantage score (e.g., including US household food security, father absence, changes in home address, family public assistance, income and education). For comparison purposes, we computed a socioeconomic composite in the same way as in the SOEP cohort instead
	Our preregistration included analyses with neighborhood SES to examine gene-by-environment interactions on self-control. Given the lack of association between polygenic indices and self-control, we did not include neighborhood SES	
(4) Polygenic indices^c^	*Polygenic Index for externalizing (PGI-EXT*) has been computed in both cohorts based on the most recent genome-wide association study (GWAS) of externalizing problems [26]. This GWAS pooled data from ~ 1.5 million people, applying a multivariate GWAS approach leveraging genetic correlations among externalizing-related measures (attention-deficit/hyperactivity disorder, problematic alcohol use, lifetime cannabis use, age at first sexual intercourse, number of sexual partners, general risk tolerance & lifetime smoking initiation). The PGI-EXT is an aggregate of the effects of observed SNPs (including 1,020,283 SNPs), weighted by their estimated effect sizes, from an independent GWAS sample. This PGI is of particular interest to our study as the score includes traits highly correlated with self-control such as ADHD, risk tolerance, problematic alcohol use, and smoking Deviating from our preregistration, the PGI for non-cognitive skills [9] was not available and therefore not included in analyses	
(5) Self-reported health	*Self-reported disease severity:* participants were asked how they would describe their current state of health on 1 item, ranging from 1 = *very good* to 5 = very *bad*, with higher scores reflecting higher self-reported disease severity	For the analyses on Health, we focused on SOEP-G as the TTP consists of children and adolescents that are generally in good health
	*Self-reported health:* participants were asked to rate across 5 items if they in the last 4 weeks experienced any limitations in life due to physical pain, physical problems or mental health problems, with 1 = *always*, to 5 = *never*, with higher scores reflecting more self-reported health In our preregistration, we did not integrate health variables (see main text for motivation). We selected health variables that previously found to be associated with the PGI-EXT [28]	
(6) Covariates	*Body Mass Index (BMI)* was computed by transforming self-reported height (in cm) and weight (in kg) in sex- and age-normed *z*-scores	
	*Smoking* was measured by self-reported tobacco use, grouping those who smoke, used to smoke or ever smoked into a smoking group versus a non-smoking group with participants who have never smoked	
	Deviating from our preregistration, we did not include substance use as a covariate as the sample sizes were too small in both samples (*n* < 5%)	
		*Pubertal development* was measured using children’s self-reports on the Pubertal Development Scale [44] assessing development across height, body hair growth, skin changes. Specific additional questions for girls included onset of menses, breast development and questions specifically for boys included, growth in body hair, deepening of voice. Pubertal development was residualized for age separately for each sex

^a^All DNAm-aging measures were residualized for array, slide, cell composition, batch (TTP only, not applicable in SOEP-G), and then standardized (mean = 0, SD = 1)

^b^All variables of interest were standardized for interpretation purposes

^c^All PGI analyses include the top principal components (PCs, 20 for SOEP-Gene, 10 for TTP) of genetic variation and genotype batch indicators

**Table 3:** Descriptives for main variables of interest in DNAm subsamples of SOEP-G and TTP

Variable	*n*	Mean	SD
*SOEP-G*			
Brief Tangney Self-Control Scale (BTS)	333	3.36	0.56
Risk Preference	829	5.58	2.28
Household Income (Euro)	1044	3318.07	1859.59
Household income/persons household	1044	1497.82	827.05
Max education household (years)	1042	13.34	2.76
Age (years)	1058	42.65	21.18
Sex	610 females		
Self-reported smoking	87 smoke		
Body Mass Index (BMI)	876	26.73	5.95
Self-reported Disease Severity	797	2.57	0.98
Self-reported Health	797	4.19	0.85
DunedinPACE	1058	1.64	0.11
PhenoAge	1058	99.15	18.81
GrimAge	1058	74.30	15.9
*TTP*			
Attention problems	1159	0.76	0.41
Impulsivity	638	10.72	3.31
Grit	702	26.06	4.30
Household Income (Euro)	733	152,303	266,504
Max education household (years)	819	17.50	2.62
Age (years)	1327	13.46	3.1
Sex	1327	647 females	
Self-reported smoking	645	58 smokers	
Body Mass Index (BMI)	1317	20.38	5.02
Pubertal development	1271	2.60	0.92
DunedinPACE	1327	1.14	0.16
PhenoAge	1327	42.78	9.57
GrimAge	1327	43.10	3.56

We compared participants who filled in the BTS to those who did not fill in this questionnaire. Those who filled in the BTS were slightly older and did not smoke, but did not significantly differ on other demographics such as education, income, BMI and gender (see Additional file 1: Table S13). For the demographics separately for older and younger participants (see “Results” section 1), see Additional file 1: Table S14. See Raffington et al. [50] for a discussion on inflated means in buccal DNA-methylation measures of biological aging

### Genotyping

#### SOEP-G

A detailed description of the genetic data in SOEP-G can be found in [28]. In short, genotyping was conducted using the Illumina Infinium Global Screening Array-24 v3.0 BeadChips. Genotypes were subject to quality control excluding participants with sex-gender mismatch, with per-chromosome missingness of more than 50%, and with excess heterozygosity/homozygosity.

The Haplotype Reference Consortium reference panel (r.1.1) for imputation was used with imputation accuracy (R2) greater than 0.1. Approximately 66% of the imputed SNPs were rare with minor allele frequencies (MAF) smaller than 0.01 and ~ 24% SNPs were common. The average imputation accuracy in the data was 0.66, with higher imputation accuracy for common SNPs (MAF > 0.05) with an average imputation accuracy of 0.92. To control for population stratification, the first 20 principal components (PCs) were computed for individuals with high genetic similarity to European reference groups, based on ~ 160,000 approximately independent SNPs with imputation accuracy ≥ 70% and MAF ≥ 0.01 [28].

#### TTP

The DNA samples were genotyped using the Illumina Infinium PsychArray at the University of Edinburgh, which assays ~ 590,000 single nucleotide polymorphisms (SNPs), insertions-deletions (indels), copy number variants (CNVs), structural variants, and germline variants across the genome. Genotypes were subjected to quality control. Briefly, samples were excluded when the call rate was < 98% and when there was inconsistent reporting between biological and self-reported sex. Variants were excluded if more than 2% of the data was missing. Untyped variants were imputed on the Michigan Imputation Server, with genotypes being phased with Eagle v2.4 and imputed with Minimac4 (v1.5.7), using the 1 K Genomes Phase 3 v5 panel as a reference panel [4]. Thresholds for minor allele frequency (MAF < 1e−3) and Hardy–Weinberg Equilibrium (HWE *p* value < 1e−6) were be applied. Imputed genotypes with poor imputation quality (INFO score < .90) were excluded.

### Preprocessing methylation data

#### SOEP-G

*Data collection* Buccal swabs and Isohelix IS SK-1S Dri-Capsules were used to collect DNA data. DNA extraction and methylation profiling were conducted at the Erasmus Medical Center in the Netherlands by the Human Genomics Facility (HuGe-F).

*DNA-methylation data* Methylation levels were assessed using the Infinium MethylEPIC v1 manifest B5 kit at 865,918 CpG sites (Illumina, Inc., San Diego, CA). All samples were from the same batch. DNAm preprocessing was conducted using Illumina’s GenomeStudio software and the packages ‘minfi,’ ‘ewastools’ and ‘EpiDISH’ in open-source *R* version 4.2.0 [3, 22, 51, 66]. Data cleaning took place in three steps.

First, 20 control metrics were generated in GenomeStudio (see BeadArray Controls Reporter Software Guide from Illumina). Samples were flagged and excluded when falling below the Illumina-recommended cutoffs, including (1) all types of poor bisulfite conversion and all types of poor bisulfite conversion background, (2) all types of bisulfite conversion background < 0.5, (3) all types of poor specificity, (4) all types of poor hybridization (excluded *n* = 43). Second, unreliable data points were identified resulting from low fluorescence intensities. Probes with only background signal in a high proportion of samples (proportion of samples with detection *p* value > .01 is > .1) and probes with a high proportion of samples with low bead numbers (proportion of samples with bead number < 3 is > 0.1) were removed. Additionally, cross-reactive probes for Epic arrays and probes with SNPs at the CG or single base extension were also removed [35, 45]. Third, we corrected for background noise and color dye bias (with ‘PreprocessNoob’ in minfi, [61], accounted for probe-type differences (with ‘BMIQ’ in minfi, [58] and estimated cell composition using robust partial correlations (with ‘HEpiDisch’ in EpiDISH). In order to call the sample a ‘buccal sample’ we set a threshold of 0.5 for epithelial cell proportions [49].

#### TTP

Methylation profiling was conducted by Edinburgh Clinical Research Facility, using the Infinium MethylationEPIC BeadChip kit (Illumina, Inc., San Diego, CA) to assess methylation levels at 850,000 methylation sites. Briefly, preprocessing was conducted with the ‘minfi’ package in R version 4.0.4 [3, 51]. Within-array normalization was performed to address array background correction, red/green dye bias, and probe type I/II correction. To correct for background correction and dye-bias equalization, we applied minfi’s “preprocessNoob” [61]. Data cleaning took place in three steps. CpG probes were excluded if (1) detection *p* > .01, (2) there were fewer than 3 beads in more than 1% of the samples, (3) they were in cross-reactive regions. Samples were excluded if (1) there was mismatch between self-reported and methylation estimated sex, (2) they showed low intensity probes as indicated by the log of average methylation and their detection *p* was > .01 in > 10% of their probes. In R we estimated composition of the immune and epithelial cell types in the samples using “BeadSorted.Saliva.EPIC” within “ewastools” in R, and surrogate variable analyses were used to correct for batch effects (3 batches) using the “combat” function in the SVA package.

### Statistical analyses

Analyses were conducted in R version 4.4.2 and Mplus 8.9 statistical software [41, 57]. To correct for dependency of observations due to clustering in families (SOEP-G for the PGI analyses) and due to repeated measures within individuals and multiple twin pairs within families (in TTP), we used a sandwich estimator to estimate cluster-robust standard errors. All models included age, gender, and an age-by-gender interaction as covariates, and all variables of interest were standardized for interpretation purposes. We report nominal *p* values taking *p* < .05 as a threshold, and additionally note if results remain significant after Benjamini–Hochberg False-Discovery-Rate method (FDR, [6]) correction. See Table 2 and Additional file 1: Table S1 for a list of preregistered analyses and measures and deviations if applicable.

### Supplementary Information

**Additional file 1: Table S1.** List of preregistered analyses (see https://osf.io/5sejf/), deviations, and results if not reported in main text. **Table S2.** Associations of self-control measures with saliva DNAm measures of biological aging measures in TTP. **Table S3.** Associations with saliva DNAm measures of biological aging and Self-control*Age interaction in TTP. **Table S4.** Associations of DNAm measures of biological aging with self-control and SES in SOEP-G. **Table S5.** Associations Between Socioeconomic disadvantage and saliva DNAm measures of biological aging in TTP. **Table S6.** Associations of DNAm measures of biological aging and self-control in SOEP-G and BMI. **Table S7.** Associations of DNAm measures of biological aging and self-control in SOEP-G and PGI-Externalizing (PGI-Ext). **Table S8.** Associations of DNAm measures of biological aging and risk-taking. **Table S9.** Associations testing the interaction effect of age*DNAm measures of biological aging on health in SOEP-G. **Table S10.** Associations between DNAm measures of biological aging and self-reported disease and health including BMI and Smoking in SOEP-G. **Table S11.** Associations between DNAm measures f biological aging and self-reported disease and health including SES in SOEP-G. **Table S12.** Indirect path estimates of DNA-methylation measures of biological aging statistically accounting for associations of self-control with health. **Table S13.** Comparing participant who filled in the Brief Tangney Self-control scale to those who did not at key demographics in SOEP-G. **Table S14.** Descriptives for main variables of interest in DNAm subsamples of SOEP-G for older and younger participants. **Figure S1.** Associations between self-control and DNA-methylation measures of biological aging in TTP. DNAm-aging measures and self-control measures are scaled. **Figure S2.** Graphical representation of indirect path estimates of DNA-methylation measures of biological aging statistically accounting for associations of self-control with health and disease. **Figure S3.** Path estimates of DNA-methylation measures of biological aging statistically accounting for associations of self-control with health and disease.

## Data Availability

The datasets used and/or analyzed during the current study are available from the corresponding author on reasonable request.

## References

[1] Achenbach TM. The Child Behavior Checklist and related instruments. In: The use of psychological testing for treatment planning and outcomes assessment. 2nd ed. Lawrence Erlbaum Associates Publishers; 1999. pp. 429–466.

[2] Arslan RC, Brümmer M, Dohmen T, Drewelies J, Hertwig R, Wagner GG (2020). How people know their risk preference. Sci Rep.

[3] Aryee MJ, Jaffe AE, Corrada-Bravo H, Ladd-Acosta C, Feinberg AP, Hansen KD, Irizarry RA (2014). Minfi: a flexible and comprehensive Bioconductor package for the analysis of Infinium DNA methylation microarrays. Bioinformatics.

[4] Auton, A., Brooks, L., Durbin, R., Garrison, E., Kang, H., Consortium, G.P (2015). A global reference for human genetic variation. Nature..

[5] Belsky DW, Caspi A, Corcoran DL, Sugden K, Poulton R, Arseneault L, Baccarelli A, Chamarti K, Gao X, Hannon E, Harrington HL, Houts R, Kothari M, Kwon D, Mill J, Schwartz J, Vokonas P, Wang C, Williams BS, Moffitt TE (2022). DunedinPACE, a DNA methylation biomarker of the pace of aging. Elife.

[6] Benjamini Y, Hochberg Y (1995). Controlling the false discovery rate: a practical and powerful approach to multiple testing. J Roy Stat Soc: Ser B (Methodol)..

[7] Bermick J, Schaller M (2022). Epigenetic regulation of pediatric and neonatal immune responses. Pediatr Res.

[8] Cobb-Clark DA, Dahmann SC, Kamhöfer DA, Schildberg-Hörisch H (2023). Self-control and unhealthy body weight: the role of impulsivity and restraint. Econom Hum Biol.

[9] Demange PA, Malanchini M, Mallard TT, Biroli P, Cox SR, Grotzinger AD (2021). Investigating the genetic architecture of noncognitive skills using GWAS-by-subtraction. Nat Genet..

[10] De Ridder DT, Lensvelt-Mulders G, Finkenauer C, Stok FM, Baumeister RF. Taking stock of self-control: a meta-analysis of how trait self-control relates to a wide range of behaviors. In: Self-regulation and self-control. Routledge; 2018. pp. 213–255. 10.1177/1088868311418749 21878607

[11] Duckworth AL (2011). The significance of self-control. Proc Natl Acad Sci.

[12] Duckworth AL, Quinn PD (2009). Short Grit Scale. J Personal Assess.

[13] Elliott ML, Caspi A, Houts RM, Ambler A, Broadbent JM, Hancox RJ, Harrington H, Hogan S, Keenan R, Knodt A, Leung JH, Melzer TR, Purdy SC, Ramrakha S, Richmond-Rakerd LS, Righarts A, Sugden K, Thomson WM, Thorne PR (2021). Disparities in the pace of biological aging among midlife adults of the same chronological age have implications for future frailty risk and policy. Nat Aging.

[14] Engelhardt LE, Church JA, Paige Harden K, Tucker-Drob EM (2019). Accounting for the shared environment in cognitive abilities and academic achievement with measured socioecological contexts. Dev Sci.

[15] Faul JD, Kim JK, Levine ME, Thyagarajan B, Weir DR, Crimmins EM (2023). Epigenetic-based age acceleration in a representative sample of older Americans: associations with aging-related morbidity and mortality. Proc Natl Acad Sci.

[16] Föhr T, Waller K, Viljanen A, Rantanen T, Kaprio J, Ollikainen M, Sillanpää E (2023). Mortality associations with DNA methylation-based biological aging and physical functioning measures across a 20-year follow-up period. J Gerontol Ser A.

[17] Finkenauer C, Buyukcan-Tetik A, Baumeister RF, Schoemaker K, Bartels M, Vohs KD (2015). Out of control: Identifying the role of self-control strength in family violence. Curr Dir Psychol Sci.

[18] Friese M, Frankenbach J, Job V, Loschelder DD (2017). Does self-control training improve self-control? A meta-analysis. Perspect Psychol Sci.

[19] Hansen TVO, Simonsen MK, Nielsen FC, Hundrup YA (2007). Collection of blood, saliva, and buccal cell samples in a pilot study on the Danish Nurse Cohort: comparison of the response rate and quality of genomic DNA. Cancer Epidemiol Biomark Prev.

[20] Harden KP, Tucker-Drob EM, Tackett JL (2013). The Texas Twin Project. Twin Res Hum Genet Off J Int Soc Twin Stud.

[21] Harvanek ZM, Fogelman N, Xu K, Sinha R (2021). Psychological and biological resilience modulates the effects of stress on epigenetic aging. Transl Psychiatry.

[22] Heiss JA, Just AC (2018). Identifying mislabeled and contaminated DNA methylation microarray data: an extended quality control toolset with examples from GEO. Clin Epigenet.

[23] Higgins-Chen AT, Thrush KL, Wang Y, Minteer CJ, Kuo P-L, Wang M, Niimi P, Sturm G, Lin J, Moore AZ, Bandinelli S, Vinkers CH, Vermetten E, Rutten BPF, Geuze E, Okhuijsen-Pfeifer C, van der Horst MZ, Schreiter S, Gutwinski S, Levine ME (2022). A computational solution for bolstering reliability of epigenetic clocks: Implications for clinical trials and longitudinal tracking. Nat Aging..

[24] Hoffmann JP (2022). Self-control, peers, and adolescent substance use: an international analysis. J Subst Use.

[25] Joyce BT, Gao T, Zheng Y, Ma J, Hwang S-J, Liu L, Nannini D, Horvath S, Lu AT, Bai Allen N, Jacobs DR, Gross M, Krefman A, Ning H, Liu K, Lewis CE, Schreiner PJ, Sidney S, Shikany JM, Lloyd-Jones D (2021). Epigenetic age acceleration reflects long-term cardiovascular health. Circ Res.

[26] Karlsson Linnér R, Mallard TT, Barr PB, Sanchez-Roige S, Madole JW, Driver MN, Poore HE, de Vlaming R, Grotzinger AD, Tielbeek JJ, Johnson EC, Liu M, Rosenthal SB, Ideker T, Zhou H, Kember RL, Pasman JA, Verweij KJH, Liu DJ, Dick DM (2021). Multivariate analysis of 1.5 million people identifies genetic associations with traits related to self-regulation and addiction. Nat Neurosci.

[27] Kirkwood TBL (2005). Understanding the odd science of aging. Cell.

[28] Koellinger PD, Okbay A, Kweon H, Schweinert A, Linnér RK, Goebel J, Hertwig R (2023). Cohort profile: genetic data in the German Socio-Economic Panel Innovation Sample (SOEP-G). PLoS One.

[29] Lei M-K, Brody GH, Beach SRH (2022). Intervention effects on self-control decrease speed of biological aging mediated by changes in substance use: a longitudinal study of African American youth. Fam Process.

[30] Levine ME, Lu AT, Quach A, Chen BH, Assimes TL, Bandinelli S, Hou L, Baccarelli AA, Stewart JD, Li Y, Whitsel EA, Wilson JG, Reiner AP, Aviv A, Lohman K, Liu Y, Ferrucci L, Horvath S (2018). An epigenetic biomarker of aging for lifespan and healthspan. Aging (Albany NY).

[31] Lo Y-H, Lin W-Y (2022). Cardiovascular health and four epigenetic clocks. Clin Epigenet.

[32] López-Otín C, Blasco MA, Partridge L, Serrano M, Kroemer G (2013). The hallmarks of aging. Cell.

[33] Loyfer N, Magenheim J, Peretz A, Cann G, Bredno J, Klochendler A, Fox-Fisher I, Shabi-Porat S, Hecht M, Pelet T (2023). A DNA methylation atlas of normal human cell types. Nature.

[34] Lu AT, Quach A, Wilson JG, Reiner AP, Aviv A, Raj K, Hou L, Baccarelli AA, Li Y, Stewart JD, Whitsel EA, Assimes TL, Ferrucci L, Horvath S (2019). DNA methylation GrimAge strongly predicts lifespan and healthspan. Aging.

[35] Mccartney DL, Hillary RF, Conole ELS, Banos DT, Gadd DA, Walker RM, Nangle C, Flaig R, Campbell A, Murray AD, Maniega SM, Valdés-hernández MDC, Harris MA, Bastin ME, Wardlaw JM, Harris SE, Porteous DJ, Tucker-drob EM, Mcintosh AM, Marioni RE (2022). Blood-based epigenome-wide analyses of cognitive abilities. Genome Biol.

[36] McCrory C, Fiorito G, Hernandez B, Polidoro S, O’Halloran AM, Hever A, Ni Cheallaigh C, Lu AT, Horvath S, Vineis P, Kenny RA (2020). GrimAge outperforms other epigenetic clocks in the prediction of age-related clinical phenotypes and all-cause mortality. J Gerontol A Biol Sci Med Sci.

[37] Meldrum RC, Barnes JC, Hay C (2015). Sleep deprivation, low self-control, and delinquency: a test of the strength model of self-control. J Youth Adolesc.

[38] Middleton LYM, Dou J, Fisher J, Heiss JA, Nguyen VK, Just AC, Faul J, Ware EB, Mitchell C, Colacino JA, Bakulski MK (2022). Saliva cell type DNA methylation reference panel for epidemiological studies in children. Epigenetics.

[39] Miller GE, Yu T, Chen E, Brody GH (2015). Self-control forecasts better psychosocial outcomes but faster epigenetic aging in low-SES youth. Proc Natl Acad Sci.

[40] Moffitt TE, Arseneault L, Belsky D, Dickson N, Hancox RJ, Harrington H, Houts R, Poulton R, Roberts BW, Ross S, Sears MR, Thomson WM, Caspi A (2011). A gradient of childhood self-control predicts health, wealth, and public safety. Proc Natl Acad Sci.

[41] Muthén B, Muthén L. Mplus: a general latent variable modeling program. 2019.

[42] Oblak L, van der Zaag J, Higgins-Chen AT, Levine ME, Boks MP (2021). A systematic review of biological, social and environmental factors associated with epigenetic clock acceleration. Ageing Res Rev.

[43] Pampel FC, Krueger PM, Denney JT (2010). Socioeconomic disparities in health behaviors. Ann Rev Sociol.

[44] Petersen AC, Crockett L, Richards M, Boxer A (1988). A self-report measure of pubertal status: reliability, validity, and initial norms. J Youth Adolesc..

[45] Pidsley R, Zotenko E, Peters TJ, Lawrence MG, Risbridger GP, Molloy P, Van Djik S, Muhlhausler B, Stirzaker C, Clark SJ (2016). Critical evaluation of the Illumina MethylationEPIC BeadChip microarray for whole-genome DNA methylation profiling. Genome Biol.

[46] Raffington L, Belsky DW (2022). Integrating DNA methylation measures of biological aging into social determinants of health research. Curr Environ Health Rep.

[47] Raffington L, Belsky DW, Kothari M, Malanchini M, Tucker-Drob EM, Harden KP (2021). Socioeconomic disadvantage and the pace of biological aging in children. Pediatrics.

[48] Raffington L, Belsky DW, Kothari M, Malanchini M, Tucker-Drob EM, Harden KP (2021). Socioeconomic disadvantage and the pace of biological aging in children. Pediatrics.

[49] Raffington L, Schwaba T, Aikins M, Richter D, Wagner GG, Harden KP, Belsky DW, Tucker-Drob EM (2022). Associations of socioeconomic disparities with buccal DNA-methylation measures of biological aging. bioRxiv.

[50] Raffington L, Schwaba T, Aikins M, Richter D, Wagner GG, Harden KP, Belsky DW, Tucker-Drob EM (2023). Associations of socioeconomic disparities with buccal DNA-methylation measures of biological aging. Clin Epigenet.

[51] R Core Team. R: a language and environment for statistical computing. 2013.

[52] Reijula S, Hertwig R (2022). Self-nudging and the citizen choice architect. Behav Public Policy..

[53] Richmond-Rakerd LS, Caspi A, Ambler A, d’Arbeloff T, de Bruine M, Elliott M, Harrington H, Hogan S, Houts RM, Ireland D, Keenan R, Knodt AR, Melzer TR, Park S, Poulton R, Ramrakha S, Rasmussen LJH, Sack E, Schmidt AT (2021). Childhood self-control forecasts the pace of midlife aging and preparedness for old age. Proc Nat Acad Sci..

[54] Robson DA, Allen MS, Howard SJ (2020). Self-regulation in childhood as a predictor of future outcomes: a meta-analytic review. Psychol Bull.

[55] Sugden K, Hannon EJ, Arseneault L, Belsky DW, Corcoran DL, Fisher HL, Houts RM, Kandaswamy R, Moffitt TE, Poulton R, Prinz JA, Rasmussen LJH, Williams BS, Wong CCY, Mill J, Caspi A (2020). Patterns of reliability: assessing the reproducibility and integrity of DNA methylation measurement. Patterns..

[56] Tangney JP, Boone AL, Baumeister RF (2018) High self-control predicts good adjustment, less pathology, better grades, and interpersonal success. In: Self-regulation and self-control. Routledge. pp. 173–212. 10.4324/9781315175775-5. 10.1111/j.0022-3506.2004.00263.x 15016066

[57] Team, R. RStudio: integrated development for R. 2020.

[58] Teschendorff AE, Widschwendter M (2012). Differential variability improves the identification of cancer risk markers in DNA methylation studies profiling precursor cancer lesions. Bioinformatics (Oxford, England).

[59] Theda C, Hwang SH, Czajko A, Loke YJ, Leong P, Craig JM (2018). Quantitation of the cellular content of saliva and buccal swab samples. Sci Rep.

[60] Tiemeijer WL, editor. The self-control effects of poverty. In: Self-control: individual differences and what they mean for personal responsibility and public policy. Cambridge University Press; pp. 117–134. 10.1017/9781009089678.007.

[61] Triche TJ, Weisenberger DJ, Van Den Berg D, Laird PW, Siegmund KD (2013). Low-level processing of Illumina Infinium DNA Methylation BeadArrays. Nucleic Acids Res.

[62] Vischer T, Dohmen T, Falk A, Huffman D, Schupp J, Sunde U, Wagner GG (2013). Validating an ultra-short survey measure of patience. Econ Lett.

[63] Wang K, Liu H, Hu Q, Wang L, Liu J, Zheng Z, Zhang W, Ren J, Zhu F, Liu G-H (2022). Epigenetic regulation of aging: implications for interventions of aging and diseases. Signal Transduct Target Therapy.

[64] Willems YE, Dolan CV, van Beijsterveldt CEM, de Zeeuw EL, Boomsma DI, Bartels M, Finkenauer C (2018). Genetic and environmental influences on self-control: assessing self-control with the ASEBA self-control scale. Behav Genet.

[65] Wong YT, Tayeb MA, Stone TC, Lovat LB, Teschendorff AE, Iwasiow R, Craig JM (2022). A comparison of epithelial cell content of oral samples estimated using cytology and DNA methylation. Epigenetics.

[66] Zheng SC, Breeze CE, Beck S, Dong D, Zhu T, Ma L, Ye W, Zhang G, Teschendorff AE (2019). EpiDISH web server: epigenetic dissection of intra-sample-heterogeneity with online GUI. Bioinformatics (Oxford, England).

[67] Zuckerman M, Aluja A. Measures of sensation seeking. In: Measures of personality and social psychological constructs. Elsevier Academic Press; 2015. pp. 352–380. 10.1016/B978-0-12-386915-9.00013-9.

